# Functional and transport analyses of *CLCN5* genetic changes identified in Dent disease patients

**DOI:** 10.14814/phy2.12776

**Published:** 2016-04-25

**Authors:** Xiaojing Tang, Matthew R. Brown, Andrea G. Cogal, Daniel Gauvin, Peter C. Harris, John C. Lieske, Michael F. Romero, Min‐Hwang Chang

**Affiliations:** ^1^ O'Brien Urology Research CenterMayo Clinic College of MedicineRochesterMinnesota; ^2^ Nephrology and HypertensionMayo Clinic College of MedicineRochesterMinnesota; ^3^ Division of NephrologyShanghai Changzheng HospitalSecond Military Medical UniversityShanghaiChina; ^4^ Physiology & Biomedical EngineeringMayo Clinic College of MedicineRochesterMinnesota; ^5^ Wayne State UniversityDetroitMichigan; ^6^ Biochemistry and Molecular BiologyMayo Clinic College of MedicineRochesterMinnesota; ^7^ Laboratory Medicine and PathologyMayo Clinic College of MedicineRochesterMinnesota

**Keywords:** Anion selectivity, pH dependence, protein trafficking, voltage clamp

## Abstract

Dent disease type 1, an X‐linked inherited kidney disease is caused by mutations in electrogenic Cl^−^/H^+^ exchanger, ClC‐5. We functionally studied the most frequent mutation (S244L) and two mutations recently identified in RKSC patients, Q629X and R345W. We also studied T657S, which has a high minor‐allele frequency (0.23%) in the African‐American population, was published previously as pathogenic to cause Dent disease. The transport properties of CLC‐5 were electrophysiologically characterized. WT and ClC‐5 mutant currents were inhibited by pH 5.5, but not affected by an alkaline extracellular solution (pH 8.5). The T657S and R345W mutations showed the same anion selectivity sequence as WT ClC‐5 (SCN ^−^>NO3^−^≈Cl^−^>Br^−^>I^−^). However, the S244L and Q629X mutations abolished this anion conductance sequence. Cell surface CLC‐5 expression was quantified using extracellular HA‐tagged CLC‐5 and a chemiluminescent immunoassay. Cellular localization of eGFP‐tagged CLC‐5 proteins was also examined in HEK293 cells with organelle‐specific fluorescent probes. Functional defects of R345W and Q629X mutations were caused by the trafficking of the protein to the plasma membrane since proteins were mostly retained in the endoplasmic reticulum, and mutations showed positive correlations between surface expression and transport function. In contrast, although the S244L transport function was significantly lower than WT, cell surface, early endosome, and endoplasmic reticulum expression was equal to that of WT CLC‐5. Function and trafficking of T657S was equivalent to the WT CLC‐5 suggesting this is a benign variant rather than pathogenic. These studies demonstrate the useful information that can be gained by detailed functional studies of mutations predicted to be pathogenic.

## Introduction

Dent disease is an X‐linked inherited disorder characterized by excessive urinary loss of protein (heavy low molecular weight in nature), calcium (hypercalciuria), and phosphate (hyperphosphaturia). Kidney stones and nephrocalcinosis are common. Despite a few recent reports of *ORCL1* mutations as a cause of Dent disease type 2 (Hoopes et al. 2005; Utsch et al. 2006), up to 85% of the cases are Dent disease type 1 caused by mutations in the *CLCN5* gene which encodes the electrogenic Cl^−^/H^+^ exchanger (transporter), ClC‐5 (Claverie‐Martin et al. 2011). More than 180 different *CLCN5* mutations have so far been reported among patients with Dent disease according to the Human Gene Mutation Database (HGMD). The major pathogenic mechanism underlying Dent disease type 1 is disruption of endosomal acidification and proximal tubular endocytosis due to the absence of functional ClC‐5 (Wellhauser et al. 2010). Endosomal acidification in the proximal tubule is essential for reabsorption of proteins, minerals, and vitamins. Chronic activation of apoptotic and inflammatory pathways in combination with nephrocalcinosis secondary to defective protein, mineral, and vitamin handling is associated with chronic kidney disease in an estimated two‐thirds of the affected patients (Claverie‐Martin et al. 2011). Therefore, early identification of ClC‐5 mutations in patients with proteinuria and/or hypercalciuria, and an improved understanding of the consequences of disease‐associated mutations on CLC‐5 function, biosynthesis, endosomal trafficking, stability, and function are vital for the development of effective targeted therapies.

## Materials and methods

### Rare kidney stone consortium dent disease registry

This study was approved by the relevant Institutional Review Boards (IRB) and Ethics Committees of Mayo Clinic College of Medicine and all participants gave informed consent. The Rare Kidney Stone Consortium (RKSC) Dent disease registry is a secure web‐based registry that enables international contributions (Lieske et al. 2005). Patients within the registry have (1) low Molecular Weight Proteinuria (at least 5 times above the upper limit of normal; (normal urinary protein excretion is <150 mg/24 h) plus (2) one of the following: a. hypercalciuria, b. kidney stones, c. nephrocalcinosis, d. hypophosphatemia, e. renal phosphate leak, f. aminoaciduria, g. glucosuria without diabetes mellitus, h. hematuria, i. renal insufficiency, j. family history with X‐linked inheritance; or (3) confirmed genetic mutation of *CLCN5* or *OCRL1*.

Patients enrolled in the RKSC Dent disease registry who consented to molecular testing had blood drawn for DNA isolation. Sanger sequencing was performed of all coding exons ±20 bp (*CLCN5*: NM_000084, *OCRL1*: NM_000276) using M13 tailed primers (Beckman Coulter). Primer sequences can be obtained by request. All Sanger chromatograms were analyzed using Mutation Surveyor v4.0.9 (Softgenetics) and identified variants were categorized as nonsense, missense, frameshifting, or inframe InDel, (insertion, duplication, deletion, insertion + deletion) typical splice (±2 bp from exon boundary) or atypical splice (>±2 bp from exon boundary). As of November 2015 a total of 77 families were screened.

### Evaluation of CLCN5 alleles

Amino acid substitutions not previously reported in HGMD were evaluated using the *in silico* prediction programs AlignGVGD (http://agvgd.iarc.fr/index.php), PolyPhen‐2 (http://genetics.bwh.harvard.edu/pph2/index.shtml), and SIFT (http://sift.jcvi.org). Additionally, for amino acid substitutions, multisequence alignments (MSA) using *CLCN5* and *OCRL1* protein orthologs of dog (*Canis lupus*), chicken (*Gallus gallus*), mouse (*Mus musculus*), rat (*Rattus norvegicus*), and zebrafish (*Danio rerio*) were used to evaluate evolutionary conservation. Amino acid substitutions were scored as pathogenic if two of three *in silico* tools predicted them as damaging, the change was evolutionarily conserved, and/or the variant segregated appropriately. MAF values were acquired by accessing data of the Exome Aggregation Consortium (ExAC), Cambridge, MA at http://exac.broadinstitute.org [November 2015] and obtaining population frequency data on variants when available.

### Molecular biology

Human wild‐type (WT) ClC‐5 (GenBank NM_000084.4) ORF was subcloned into the pGEMHE expression vector for *Xenopus laevis* oocytes expression, or into the peGFPc2 expression vector for expressing in HEK293 cells. Four representative ClC‐5 mutation variants (T657S, R345W, S244L, and Q629X, Table 2) were generated by site‐directed mutagenesis using the QuickChange site‐directed mutagenesis kit (Stratagene, La Jolla, CA). The HA epitope (YPYDVPDYA) was introduced into the extracellular loop of ClC‐5 in pGEMHE vector between transmembrane domain B and C (Dutzler et al. 2002). All constructs were fully sequenced.

### Expression in Xenopus. laevis Oocytes

Capped cRNA were synthesized *in vitro* from wild‐type and mutant ClC‐5 expression vectors linearized with MluI using the T7 mMessage mMachine Kit (Ambion, Austin, TX). Frogs were housed and cared for in accordance and approval of the Institutional Animal Care and Use Committee of the Mayo Clinic College of Medicine. Defolliculated *X. laevis* oocytes were injected with 10 ng of the specific cRNAs. The oocytes were then kept at 16°C in OR3 media.

### Electrophysiology

Two or three days after injection, two‐electrode voltage clamp experiments were performed at room temperature using an OC‐725C voltage clamp (Warner instruments, Hamden, CT) and Heka software (Wiesenstrasse, Germany). Currents were recorded in either ND96 solution (96 mmol/L NaCl, 2.0 mmol/L KCl, 1.8 mmol/L CaCl_2_, 1.0 mmol/L MgCl_2_, 5.0 mmol/L HEPES, pH 7.5) or experimental solutions with iso‐osmotic ion replacements. Currents were recorded in response to a voltage protocol consisting of 20 mV steps from −120 mV to +80 mV during 800 msec/step from a holding potential of −60 mV.

### Determine oocyte surface ClC‐5 expression by chemiluminescent immunoassay

An hemagglutinin (HA) tag was engineered and added to the extracellular loop of *CLC5* between transmembrane domains B and C (Dutzler et al. 2002) to track its intracellular trafficking. Previous studies have shown that the HA epitope tag at this location does not interfere with ClC‐5 function (Schwake et al. 2001).

Surface protein labeling of oocytes expressing CLC‐5 and CLC‐5 mutants was performed according to the previously established method described by our group (Chang et al. 2008). At 4°C, oocytes were fixed with 4% paraformaldehyde in ND96 for 15 min, washed, and incubated in 1% bovine serum albumin (BSA)‐ND96 blocking solution for 30 min. Oocytes were labeled with a primary antibody (1:200 dilution, monoclonal rat‐*α*‐HA 1° antibody [Roche]) for 60 min and then with a secondary antibody (1:2000 dilution, horseradish peroxidase conjugated goat‐*α*‐rat IgG [Jackson Labs]) for 30 min in 1% BSA‐ND96 blocking solution. Labeled oocytes were washed several times and incubated in ND96 for 10 min before exposure to 50 *μ*L of the premixed SuperSignal ELISA Femto substrate solution (Pierce Scientific) at room temperature. Chemiluminescence was measured from single oocytes in a microcentrifuge tube using a TD‐20/20n luminometer (Turner BioSystems).

### Cell culture and transfection

HEK293 cells were grown in Dulbecco's modified Eagle's medium (DMEM) (GIBCO, Invitrogen, CA) supplemented with 10% fetal bovine serum, penicillin (100 IU/mL), and streptomycin (100 mg/mL) at 37°C in 5% CO_2_. The cells were transiently transfected with eGFP‐tagged WT ClC‐5 and mutants using Fugene 6 according to the Manufacturer's instructions (Roche Diagnostics).

### Cellular localization

WT and mutant ClC‐5 transfected HEK293 cells were incubated on Falcon six‐well cell culture plates and stained with organelle‐specific fluorescence probes. Early endosome and endoplasmic reticulum were transduced with RFP‐actin using BacMam reagent (Invotrogen, CellLight^®^ Reagent BacMam 2.0). The reagent was directly added to the cell suspension when the cell density was 1.5 × 10^5^ cells/mL. Cells were then analyzed with a Zeiss Observer Z1/ApoTome microscope (Carl Zeiss, Germany) after incubation at 37°C with 5% CO_2_ for 16 h. The cell membrane was stained with 5 *μ*g per well of Cellmask Deep Red plasma membrane stain (Life Technologies). Cells were incubated for 5 min with the reagents at 37°C and then washed three times with DMEM. Labeled cells were analyzed with the Zeiss ApoTome microscope.

### Statistics

Analyses were completed using JMP^®^ version 9.0.1 (SAS Institute Inc.). All values are expressed as the mean and standard error of the mean (M ± SEM). n indicates the number of experiments. A value of *P* < 0.05 was considered statistically significant.

## Results

There were 193 reported pathogenic mutations of *CLCN5* in the literature as of November 2015. Of these mutations, S244L is one of the most common (Hoopes et al. 2004; Tosetto et al. 2006; Mansour‐Hendili et al. 2015; Pusch and Zifarelli 2015). Q629X and R345W are both novel mutations that were detected in RKSC patients, although Q629X has also since been reported in a Japanese cohort (Sekine et al. 2014). Both R345W and T657S were detected in the ExAC database; however, T657S was previously reported as pathogenic in a Dent disease patient (Hoopes et al. 2004) and was found with a high minor‐allele frequency, which was most marked in African Americans (MIF MAF 0.23%) (Table 1). Thus, these four mutations were selected for detailed physiologic characterization: two because they were apparently novel, one because it might have a link to renal disease in African Americans, and the final as a common pathogenic variant to serve as a positive control.

**Table 1:** Minor‐allele frequency in populations

Mutation	Database	MAF% entire population	MAF% African American	MAF% Latino
R345W (c.1033C>T) a	ExAC:	0.0034 (T = 3/C = 87505)		
T657S (c.1970C>G) b	ExAC:	0.0282 (G = 19/C = 67481)	0.2300 (G = 17/C = 7405)	0.0274 (G = 2/C = 7295)
Q629X (c.1885C>T)	ExAC:	NA		
S244L (c.731C>T)	ExAC:	NA		

a detected only in Non‐Finnish European population.

b detected mainly in African‐American population plus twice in Latino population.

As shown in Table 2, the RKSC Dent Registry patients carrying the selected mutations had variable clinical manifestations. One patient with the S244L mutation had mild hypercalciuria, nephrocalcinosis, and chronic kidney disease (eGFR 22.2 mL/min/1.73 m^2^), whereas another patient with the S244L change had normal kidney function. The two patients with the Q629X mutation are brothers. One brother had kidney stones and rickets with normal kidney function, whereas the other brother had neither urinary stones nor bone disease. The patient with the R345W mutation had impaired kidney function (eGFR 35.3 mL/min/1.73 m^2^) without other symptoms. As noted above the T657S mutation was reported in a previous publication with no detailed description of the patient's clinical features (Hoopes et al. 2004).

**Table 2:** Clinical manifestation of the patients with Dent disease

Pedigree	Mutation	LMWP	Rickets	Hypercalciuria	Kidney stone	NC	Latest eGFR
Ia	Q629X	Y	Y	Y	Y	N	121.7
Ib	Q629X	Y	N	Y	N	N	104.5
II	S244L	Y	N	Y	N	Y	38.8*
III	S244L	Y	N	N	N	N	128.0
IV	R345W	Y	N	N	N	N	35.3

Patient a and b are brothers. All patients have LMWP, but other symptoms are quite variable. * estimated by creatinine after kidney transplantation.

LMWP, low molecular weight proteinuria; NC, nephrocalcinosis; eGFR, estimated glomerular filtration rate (normal at 90–120 mL/min/1.73 m^2^).

### ClC‐5 transport function

**Figure 1:**
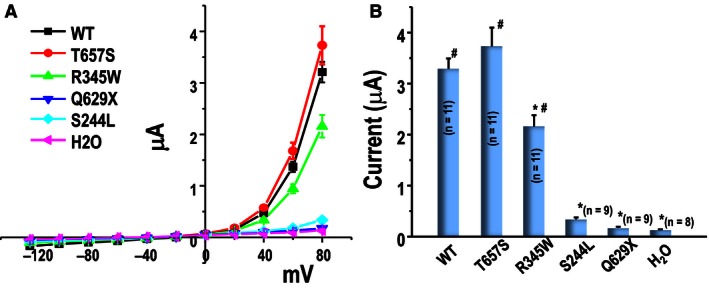
Voltage‐gated outward currents of WT and mutant ClC‐5 expressed in Xenopus oocytes. Panel (A): Current–voltage relationships obtained in ND96 solution of WT and mutant ClC‐5. Panel (B): Currents at +80 mV of WT, mutant ClC‐5 and water‐injected control. Each data point represents the mean ± SEM for at least nine oocytes from three different oocyte batches. ^#^ *P* < 0.001 is the difference between WT or mutant ClC–5 versus water‐injected oocytes. *P < 0.001 is the difference between water‐injected oocytes or mutant ClC‐5 versus WT ClC‐5.

**Figure 2:**
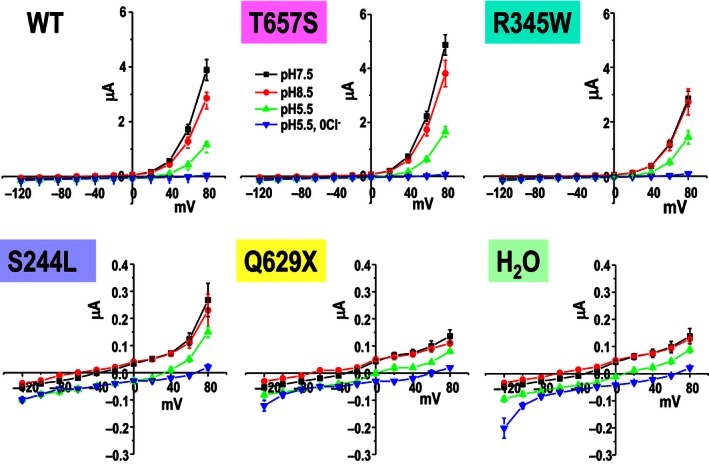
pH dependence of WT and mutant ClC‐5. ClC‐5 currents were inhibited by lowering the extracellular pH (pH 5.5) but not affected by elevating extracellular pH (pH 8.5).

**Figure 3:**
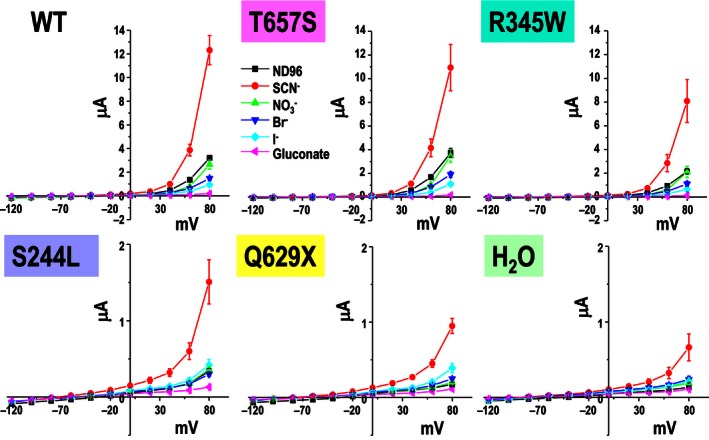
Anion selectivity of WT and mutant ClC‐5. T657S and R345W showed the same anion selectivity sequence as WT CLC‐5 (SCN ^−^>NO_3_^−^≈Cl^−^>Br^−^>I^−^). Thiocyanate (SCN ^−^) has highest anion conductance among all the anions tested for WT and mutant ClC‐5.

Wild‐type and selected human CLC‐5 mutants were injected into oocytes to examine their electrophysiological transport properties. Strongly outward rectifying currents were recorded for WT CLC‐5. The T657S mutants were found to have a similar current to WT ClC‐5. In contrast, oocytes expressing the R345W mutant displayed a significant reduction by 34.5% in current amplitude (*P* = 0.0001, Fig.****** 1). The Q629X and S244L mutants showed current reductions of 94.7% and 89.7%, respectively, which were not significantly different from those observed in water‐injected control oocytes. As previously reported (Picollo and Pusch 2005), WT and mutant ClC‐5 currents were inhibited by an extracellular acidic pH (ND96, pH5.5) compared to an alkaline solution (ND 96, pH 8.5) (Fig.****** 2). Anion selectivity experiments (Fig.****** 3) revealed that WT CLC‐5 displayed a SCN^‐^>NO3^−^≈Cl^−^>Br^−^>I^−^ conductance sequence. The T657S and R345W proteins had the same anion selectivity sequence as WT ClC‐5. However, the S244L and Q629X mutants lost the WT anion conductance sequence as their current magnitudes were too low to distinguish the anion selectivity. For the only exception was the anion SCN^−^, Thiocyanate, which continues to have the highest anion conductance among all the anion tested for WT and mutant ClC‐5.

### ClC‐5 Cellular localization

**Figure 4:**
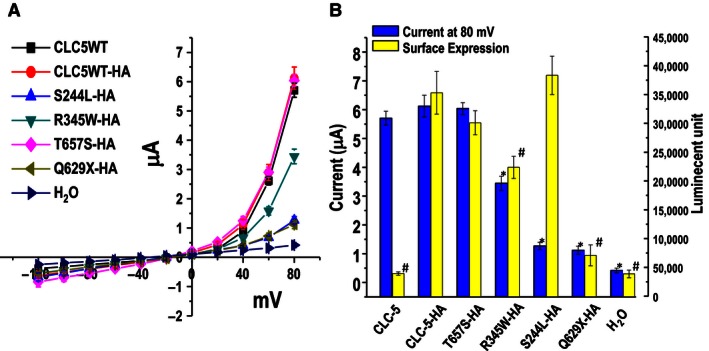
Function and surface expression of HA‐tagged WT and mutant CLC‐5 expressed in *Xenopus* oocytes. (A) Current–voltage relationships obtained in 104 mmol/L Cl^−^ (ND 96). (B) Averaged currents recorded at +80 mV (blue bars) and surface expression (yellow bars) of CLC‐5 mutants. Data shown are means ± SE and collected from at least three different batches of oocytes. *P < 0.05, current is significantly different from CLC‐5 WT HA. ^#^ *P* < 0.05, expression is significantly different from CLC‐5 WT HA.

To further elucidate the mechanism of altered currents, cell surface localization was quantified by expressing extracellular HA‐tagged WT and mutant CLC‐5 in oocytes and eGFP‐tagged proteins in HEK cells. Voltage clamp recordings confirmed that the HA and eGFP epitope did not interfere with ClC‐5 function (Fig. 4). The chemiluminescent signals (HA surface expression) did not significantly differ between the T657S, S244L, and WT ClC‐5 proteins. R345W surface protein expression was reduced compared to WT (P < 0.05). No surface expression was detected for the Q629X mutant. Furthermore, current magnitude and cell membrane protein expression was positively correlated in oocytes expressing T657S, R345W, and Q629X mutations (Fig. 4). In contrast, although the electrical activity of S244L was reduced, its cell surface protein expression did not significantly differ from the WT.

**Figure 5:**
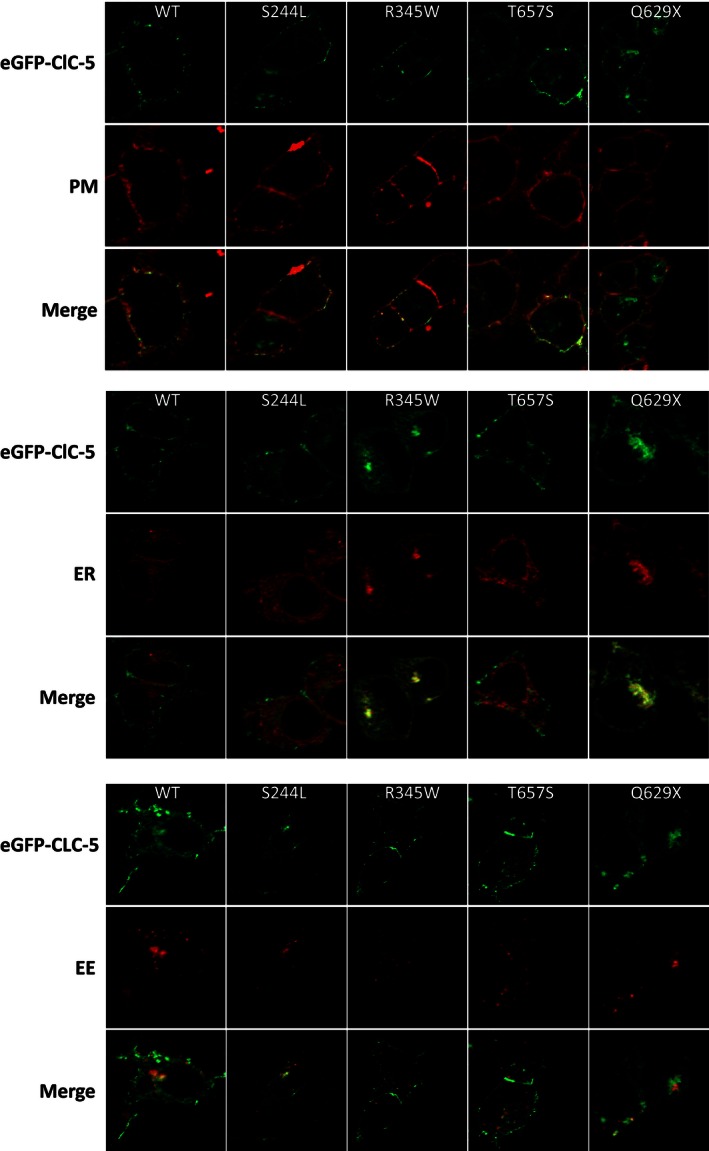
Subcellular localization of WT and mutant ClC‐5 in transfected HEK293 cells. Plasma membrane (PM; top panel) was stained with Deep Red plasma membrane stain. Endoplasmic reticulum (ER; middle panel) and early endosome (EE; bottom panel) were transduced with RFP‐actin fluorescence probes. ClC‐5 expression and organelles were detected by green and red fluorescence, respectively. The yellow fluorescence indicated the overlap of ClC‐5 protein and the organelles.

Subcellular localization of eGFP‐tagged WT and mutant CLC‐5 proteins were also measured in transiently transfected HEK‐293 cells using organelle‐specific fluorescence probes. As Figure 5 demonstrates, WT ClC‐5 was expressed both in early endosomes and on the cell surface. WT ClC‐5 also weakly colocalized with the endoplasmic reticulum. Similarly, both the T657S and S244L mutants colocalized with the plasma membrane, early endosomes, and endoplasmic reticulum and were indistinguishable from WT ClC‐5. In contrast, localization of the R345W mutant to the plasma membrane and early endosome were both much weaker than for WT CLC‐5. Instead, most of the R345W and all the Q629X proteins were retained in the endoplasmic reticulum.

## Discussion

Previous studies suggested several mechanisms that might explain loss of ClC‐5 function in patients with Dent disease (Grand et al. 2009, 2011; Smith et al. 2009). In this study, we carefully investigated the functional effects of four CLC‐5 mutations, including three missense mutations (S244L, R345W, T657S) and one nonsense mutation (Q629X). The S244L and R345W mutations are predicted to affect protein function by all *in silico* tools, including Align GVGD, Polymorphism Phenotyping v2 (PolyPhen‐2) and SIFT websites. Consistent with these predictions, the voltage clamp experiments verified that the currents of both mutants were reduced or deficient. However, *in silico* predictions for the T657S variant differed since PolyPhen‐2 predicted this change was benign, whereas the Align GVGD and SIFT predicted it was deleterious. Our studies suggest that the PolyPhen‐2 tool made a correct prediction in this instance.

Previous studies suggest that *CLCN5* missense mutations produce three kinds of functional abnormalities (Wu et al. 2003; Grand et al. 2009). The first group results in retention of CLC‐5 in the endoplasmic reticulum with no electrical activity. These mutations usually affect formation of the dimer interface and therefore lead to improper folding and rapid degradation (Wu et al. 2003). The second group does not affect localization but impacts CLC‐5 function. Previous studies suggested that a “gating glutamate (E211)” or “proton glutamate (E286)” were responsible for the chloride and proton transport functions of CLC‐5 (Zdebik et al. 2008; Grand et al. 2011). Neutralization of “gating glutamate” (E211A) converted ClC‐5 into a pure anion conductor, whereas mutation of the “proton glutamate” (E268A) abolished both chloride and proton transport. Nevertheless, both mutations did not affect localization to the plasma membrane. S244L is one of the most common mutations found in Dent 1 patients in the literature to date. This mutation appears to severely reduce currents but not affect its cellular distribution as found in the current and previous studies (Grand et al. 2011). The S244L mutation is located at helix G, which is between the “gating glutamate” and “proton glutamate”. The exact reason(s) why this localization of the S244L mutation results in these functional affects is still not clear. More studies are needed to determine if the S244L mutation would affect the gating/proton glutamate and thus disrupt chloride and proton transport.

The third group of *CLCN5* mutations results in altered subcellular distribution and defective electrical activity. Moreover, the current reduction correlated positively with decreased surface expression. These mutations are located at the periphery of the interface, which should cause relatively minor protein‐folding defects (Grand et al. 2011). Our results with the R345W mutated protein which is located in helix J were consistent with this functional feature.

The T657S mutant is located around PY‐motif (667PPLPPY672) in the intracellular C‐terminus. When expressed in *Xenopus* oocytes, ClC‐5 point mutations destroying the PY domain were found to increase channel activity due to its markedly prolonged plasma membrane retention time (Schwake et al. 2001). Similarly, our functional study also showed that the T657S mutant displayed a slightly higher electrical current (not statistically different) compared to WT with a normal subcellular distribution. The T657S mutant, previously reported by Hoopes et al. (Hoopes et al. 2004), was the only known *CLCN5* variant found in the ExAC database and had a microallele frequency of 0.23% in the African‐American population. Since African Americans are in general at greater risk for chronic kidney disease (e.g., African American Study of Kidney Disease and Hypertension (AASK) and Chronic Renal Insufficiency Cohort (CRIC) (Sika et al. 2007; Parsa et al. 2013), this finding was of potential interest. However, Rickheit and coworkers (Rickheit et al. 2010) recently generated a knock in mouse in which the PY‐motif was destroyed by a point mutation (ClC‐5^Y762E^) and demonstrated these knock‐in mice displayed neither low molecular weight proteinuria nor hyperphosphaturia. Neither cell surface expression nor endocytosis was functionally changed in proximal tubular cells from the knock in mice. Similarly, our studies found that the T657S change did not affect CLC‐5 protein localization or function. Therefore, we conclude that the T657S variant is likely nonpathogenic and is likely a polymorphism instead, although further investigation is likely warranted, given the relatively common frequency of this allele in the African‐American population. This study also highlights the importance of careful *in vivo* and *in vitro* study to confirm the pathogenic nature of missense mutations.

The Q629X change results in a nonsense mutation that is predicted to induce a truncated ClC‐5 protein lacking 117 amino acids of the C‐terminus and deletion of a cystathionine beta‐synthase (CBS) domain (Meyer et al. 2007; Wellhauser et al. 2010). Our results demonstrated that the Q629X protein was retained in the endoplasmic reticulum and thus has no surface electrical activity. Lack of the CBS domain presumably interferes with proper folding of the C terminus which is necessary for passage to the endoplasmic reticulum (Carr et al. 2003; Meyer et al. 2007; Zifarelli and Pusch 2009). Even the shortest reported truncation (R718X) results in ER retention, suggesting that the C‐terminal region is important for proper folding of ClC‐5 (Grand et al. 2009).

In summary, patients with Dent 1 disease have a variety of clinical manifestations that seem not directly linked to the specific *CLCN5* mutation. In addition, the pathogenic nature of given *CLCN5* genetic changes cannot always be predicted using available *in silico* tools. Thus, in order to determine causality, *in vitro* and/or *in vivo* studies are ultimately needed to verify the pathogenic nature of each missense change identified in a patient with a Dent phenotype. Gene expression analyses of proximal tubular cells from a *CLCN5* knock out mouse model found that several genes (e.g., Clcn3, Slc34a3, Slc9a10, etc.) that encoded ion transport proteins were either up or downregulated(Guggino 2009). Given the variable phenotype of Dent 1 patients, even with the same causative mutation, future studies to determine if other regulatory proteins or transporters exist that modify the effects of specific *CLCN5* mutations seem warranted.

## Conflict of Interest

None declared.
